# Nervous System of *Periplaneta americana* Cockroach as a Model in Toxinological Studies: A Short Historical and Actual View

**DOI:** 10.1155/2012/143740

**Published:** 2012-05-14

**Authors:** Maria Stankiewicz, Marcin Dąbrowski, Maria Elena de Lima

**Affiliations:** ^1^Biophysics Laboratory, Institute of General and Molecular Biology, N. Copernicus University, 87-100 Torun, Poland; ^2^Departamento de Bioquímica e Imunologia, Instituto de Ciências Biológicas, Universidade Federal de Minas Gerais, Belo Horizonte, 31270-901 MG, Brazil

## Abstract

Nervous system of *Periplaneta americana* cockroach is used in a wide range of pharmacological studies, including electrophysiological techniques. This paper presents its role as a preparation in the development of toxinological studies in the following electrophysiological methods: double-oil-gap technique on isolated giant axon, patch-clamp on DUM (dorsal unpaired median) neurons, microelectrode technique *in situ* conditions on axon in connective and DUM neurons in ganglion, and single-fiber oil-gap technique on last abdominal ganglion synapse. At the end the application of cockroach synaptosomal preparation is mentioned.

## 1. Introduction

**Figure 1:**
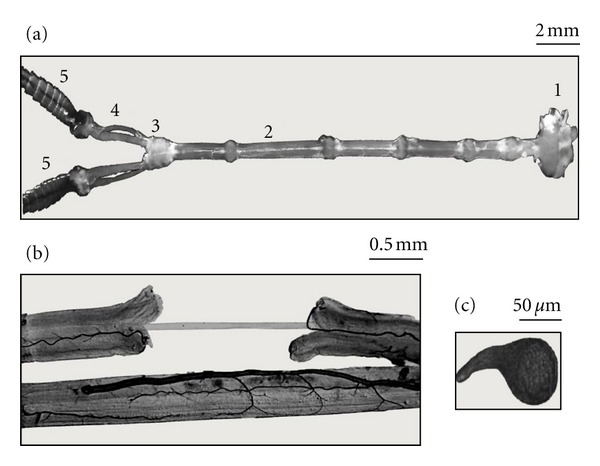
Cockroach (*Periplaneta americana*) nervous system. (a) Isolated abdominal nerve cord with last thoracic (1) ganglion and cercal nerves (4) linked to cerci (5). Giant axon is isolated from one connective between the 4th and the 5th ganglion (2). DUM neurons and cercal nerve-giant interneuron synapses are located in last abdominal ganglion (3). (b) Isolated giant axon dissected from one connective, accompanied by the second connective which protects the axon when the preparation is transferred to experimental chamber; axon diameter ~50 *μ*m. (c) Isolated DUM neuron.

The cockroach, especially the *Periplaneta americana *species, is recognized as a very useful model in neurobiological studies [1]. The field of toxinology owes much to the use of various nervous preparations obtained from this insect. *Periplaneta americana* represents an excellent model applied in different pharmacological methods, especially in electrophysiology, which plays a vital part in most of research activity in toxinology. It may be based on using natural and “artificial” preparations, as for example the transfected *Xenopus* oocytes. A wide range of nervous functions have been described on the basis of studies on various parts of cockroach nervous system (Figure 1(a)) and the experiments can be performed on natural models. Biophysical principles of the nervous system function in insects are much the same as in mammals. In both groups of animals similar neurotransmitters can be found, although their distribution varies. Thus the observations made on the cockroach can nearly be applied in vertebrates. On the other hand, some arthropod neurotoxins show high selectivity to insect nervous system and they are considered as potent bioinsecticides. Cockroach model has been largely used for the description of their mode of action. In the following paragraphs, various electrophysiological methods using cockroach preparations will be presented, along with their contribution to the development of toxinology.

## 2. Giant Axon and Single-Fiber Double-Oil-Gap Method

**Figure 2:**
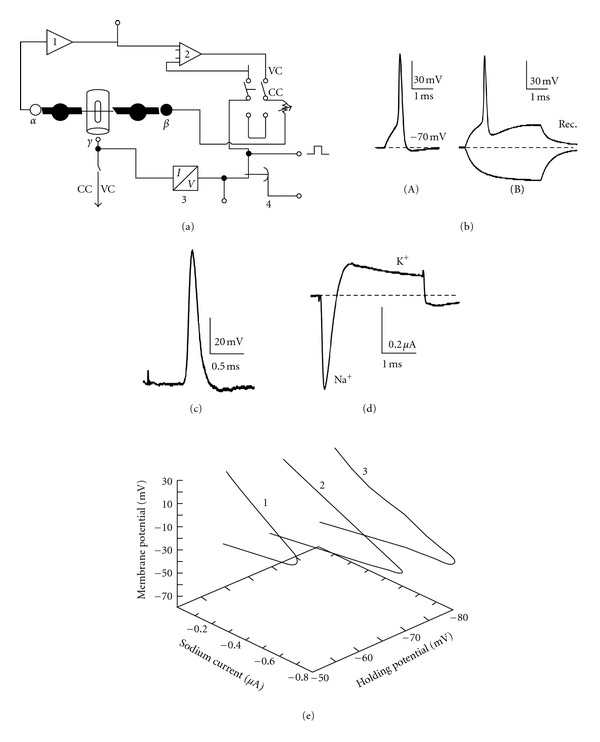
Single-fiber double-oil-gap method on isolated giant axon. (a) Electronic arrangement of technique for current- and voltage-clamp recordings (CC and VC, resp.)—according to [4]. *α* and *β* are the lateral (recording and stimulating) electrodes immersed in 180 mM KCl—they have contact with the cut ends of axons in connective and represent intracellular electrodes; *γ* electrode is plunged in physiological saline (with the tested substances)—it has contact with the extracellular side of the isolated axon. Amplifier 1 is a high-input impedance, negative capacitance amplifier, 2—high-gain differential amplifier, 3—current-to-voltage converter; 4—analogue compensator for leakage and fast and slow capacitive currents. In CC *γ* electrode is grounded and resistor = 3 MΩ. (b)(A) Action potential evoked by a 0.5 ms depolarizing current pulse. (b)(B) The effect of long symmetrical current pulses: the hyperpolarizing one—used to estimate the passive axonal membrane properties and the depolarizing one—used to observe the membrane rectification (Rec.). (c) Action potential recorded from *in situ* giant axon using microelectrode. (d) Total current recorded under voltage pulse from holding potential −70 mV to −10 mV; K^+^—potassium component, Na^+^—sodium component. (e) Voltage-dependence of sodium current recorded at various holding potentials: −1 : −60, 2 : −70 and 3 : −80 mV. The points (not shown) for curves are mean values from 5 experiments performed in control conditions. Note that at holding potential of −60 mV, about 50% of Na current is inactivated.

Cell body and the dendritic tree of the *P. americana *giant interneurons, located in the last abdominal ganglion, have been well identified for a long time. They possess unmyelinated, very long (1.5–2 cm), large diameter (up to 50 *μ*m) axons. The axons, being surrounded by glial Schwann cells, can be isolated manually (Figure 1(b)) under microscope, and their activity can be observed using the double-oil-gap method—a refined electrophysiological technique—Figure 2(a) [2–5]. Such axonal preparation exhibits simple bioelectrical properties; one type of voltage-dependent sodium and potassium (Kdr) channels can be found; they are responsible for generating short (0.5 ms) action potentials. Double-oil-gap technique permits, in current-clamp, to evoke large (up to 100 mV) axonal action potentials (Figure 2(b)(A)), to follow their evolution and to control the resting potential as well as the level of local response. Passive characteristics of axonal membrane (resistance and capacitance) can be analyzed using longer (e.g., 5 ms) hyperpolarizing pulses (Figure 2(b)(B)); the application of long depolarizing pulses allows to observe delayed rectification (Figure 2(b)(B)).

In voltage-clamp configuration, depolarizing voltage pulses induce a short, inwardly directed, tetrodotoxin-sensitive sodium current and delayed, noninactivating outward potassium current (Figure 2(d)), most sensitive to 3,4 diaminopyridine [6]; hyperpolarizing pulses are used to record the leak current. At holding potential of −60 mV, about 50% of sodium current is inactivated; this inactivation is completely removed when the HP reaches −80 mV and, in these conditions, a larger current can be observed, similarly to what has been reported by Pichon [2] (Figure 2(e)). Double-oil-gap method has several advantages: (1) the recordings are stable and long-lasting (up to 1 hour), (2) various experimental protocols in current-clamp as well in voltage-clamp can be applied, (3) the quantity of the tested molecules can be very small (e.g., 0.2 mL 10^−7^ M in the case of a highly active substance), (4) it is inexpensive. However, the preparation of an isolated axon is a sophisticated procedure and requires much experience.

The activity of giant axon can also be recorded in *in situ* conditions in the whole nerve cord [7]. In such case, axon is left on its place in nervous chain, stays in contact with others axons and its microenvironment is much less changed compared to isolated axon. After one connective is desheathed, the giant axons become accessible for microelectrodes. Introducing them allows to record the exact axonal resting potential in its *semi*-physiological microenvironment. Extracellular stimulation, even performed from some mm distance, evokes action potentials similar in size and amplitude to action potential recorded from the isolated axon (Figure 2(c)), however, long-pulse stimulation can only accelerate the generation of action potential. The accessibility of the axon *in situ* to the tested molecules is much more limited, however, their effect can be considered more physiological in such conditions.

Double-oil-gap method has been used in toxinological studies for many years. The long history of anti-insect scorpion toxins started with this technique, when in the 1970–80s the toxins purified from North African scorpions (*Androctonus australis* Hector—AaH IT and *Buthus judaicus—*Bj IT) by Professor Eliahu Zlotkin and collaborators from the Hebrew University of Jerusalem, Israel, and Faculté de Médecine, Marseille, France [8], were tested for the first time on cockroach isolated giant axon by electrophysiologist Professor Marcel Pelhate, at Angers University, France [9, 10]. Toxicity tests and biochemical studies, especially by means of binding assays, established the specificity of this toxin, which has been confirmed by electrophysiological experiments. In the subsequent years, the story developed with the application of more and more modern methods and presently many details of arthropod toxins' selectivity to insects are known [11–13]. Their applicability as bioinsecticides is now under consideration [12, 14, 15].

**Figure 3:**
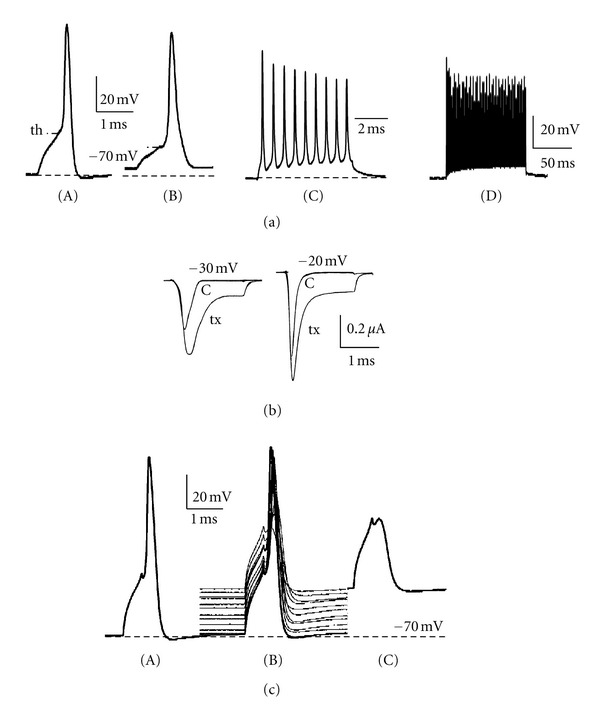
The effects of beta group of scorpion toxins on axonal bioelectrical activity. (a) The effect of Bj-xtrIT—a recombinant excitatory anti-insect toxin from *Buthus judaicus* scorpion: (A) control action potential; (B) action potential recorded from the same axon in 10 min of toxin action (10^−7^ M)—note much lower threshold (th) for action potential generation and a slight membrane depolarization; (C) repetitive activity evoked after artificial repolarization of axonal membrane and stimulation with single, short (0.5 ms) current pulse; (D) long duration, high frequency repetitive activity observed after the artificial membrane hyperpolarization to −80 mV—activity was evoked by a single, short stimulation. (b) Bigger and prolonged sodium current recorded after 10 min of Bj-xtrIT presence (tx, 10^−7^ M) compared to control (c); currents were elicited with voltage pulses from HP = −70 mV to −30 and −20 mV. (c) Axonal activity modified by Lqh IT2 (10^−6^ M)—anti-insect depressant toxin. (A) control action potential and (B) the progressive decrease of its amplitude together with the membrane depolarization induced by toxin; (C) block of action potential generation observed after 15 min of toxin action.

Scorpion toxins, active on sodium channels, are divided into two large groups: *alpha* and *beta* toxins, modifying mainly the channels' inactivation and activation, respectively [12, 13, 16, 17]. Among *alpha* toxins, an *alpha*-like subgroup has been established [18, 19]. The insect-selective toxins, depressant and excitatory, are *beta* toxins. Several toxins related to these groups were tested and their mode of action was determined using the single-fiber oil-gap method. It helped to discriminate between excitatory and depressant groups of toxins. The most distinctive features of axonal bioelectric activity modification induced by excitatory toxins (AaH IT1 from *Androctonus australis* Hector, Bj IT1 from *Buthotus judaicus*—[9, 10, 20]; Bm 32-VI and Bm 33-I from *Buthus martensi*—[21]; are a slight depolarization, a decrease in threshold for action potential generation (Figure 3(a)(A) and (B)), and the repetitive activity instead of a single response to current stimulation (Figure 3(a)(C)). The ability of the axon to generate such bursting discharges manifested itself especially when it was artificially repolarized or hyperpolarized (Figure 3(a)(D)). Exalted axonal excitability resulted from the increase in sodium current amplitude at negative membrane potentials and its prolongation observed under voltage pulse (Figure 3(b)). The analysis of sodium current voltage dependence revealed a shift in sodium current voltage dependence towards more negative membrane potentials [20]. In toxicity tests, these toxins caused an immediate contraction paralysis of fly larvae and a quick excitatory “knock-down” effect on locusts [8, 10], which remains consistent with the observations done in electrophysiological experiments.

The name “depressant toxins” comes from the flaccid paralysis they cause in fly larvae. The toxins within this group (Bj IT2—[10]; toxin from *Scorpio maurus palmatus* venom—[22]; Bot IT3 and BoT IT4 from *Buthus occitanus tunetanus*—[23, 24]; BmK ITa and BmK ITb from *Buthus martensi* Karsch—[23]; Lqh IT2—[25] evidently increase sodium permeability at resting potential, inducing relatively fast depolarization and distinct decrease in action potential amplitude (Figure 3(c)(A) and (B)), eventually blocking the conduction (Figure 3(c)(C)). In voltage-clamp experiments, the lowering of peak sodium current was observed, however, along with a development of a constant current, at holding potential equal to axonal resting potential, that is, −60 mV.

 There have also been cases of finding toxins of which the effects on axonal electrophysiological activity appear intermediate between those of excitatory and depressant groups, for instance: BcTx1 from East African scorpion *Babycurus centrurimorphus* [26] or BoT IT2, which, moreover, induced a new, unusual current with slow activation/deactivation kinetics [23, 24, 27]. Bot IT2 is insect-selective; its binding site is similar to that one of AaH IT1 excitatory toxin, but amino acid sequence resembles the ones found in depressant toxins [23, 24]. Similar modification of axonal bioelectrical activity by Bot IT2 has been evidenced using a toxin from the venom of ant, *Paraponera clavata*, also tested on the cockroach isolated axon [28].

The comparative analysis of electrophysiological effects of several excitatory and depressant toxins suggests that the discrimination between these two groups of toxins is not always easy and certainly not possible only on the basis of bioelectrical activity modifications. Parallel biochemical and structural studies are absolutely required. As mentioned above, these two groups of toxins belong to one very large group of *beta* toxins that bind to receptor 4 on sodium channel [13, 14, 17].

Toxin VII from South American scorpion, *Tityus serrulatus*, is a typical *beta* toxin with no selectivity to any group of organisms [29, 30]. Its effect on cockroach axon can also be described as intermediate between those of excitatory and depressant toxins. In the presence of the toxin, resting depolarization was observed jointly with a tendency to repetitive discharges. Sodium current was activated at more negative potential than normally; it was prolonged, but holding current development was slower than in the case of depressant toxins [31].

**Figure 4:**
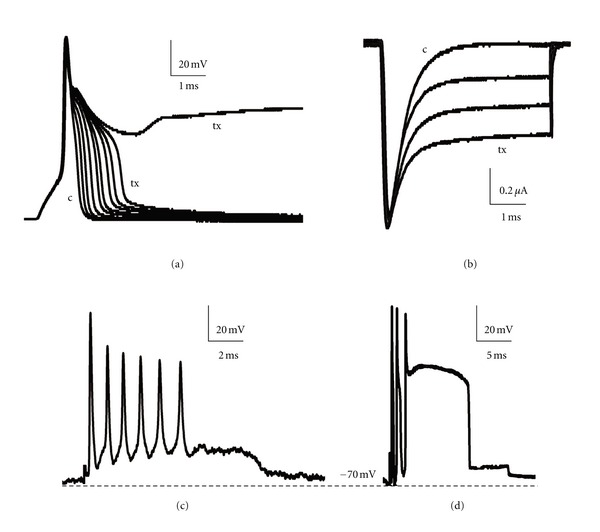
(a) The effect of Lqh*α*IT (10^−7^ M)—recombinant anti-insect *alpha* toxin from *Leiurus quinquestriatus hebraeus* venom on axonal action potential; c—control action potential, tx—progressive increase of action potential duration until *plateau* action potential under toxin. (b) sodium current prolonged until the end of depolarizing pulse in toxin presence (tx). (c) Repetitive discharges induced by Lqh*α*IT (5 × 10^−6^ M) recorded from the axon in connective (*in situ*), using microelectrode technique; connective was stimulated by extracellular silver electrodes through an isolated unit. (d) *Plateau* action potential generated sometimes by axon *in situ* in toxin presence.

Scorpion *alpha* toxins bind to the receptor site 3 on sodium channel and inhibit the channel's inactivation [12]. In voltage-clamp experiments, short sodium current is prolonged during depolarizing voltage pulse (Figure 4(b)); potassium current is not modified. In current-clamp, short action potentials are extended and transformed into *plateau* action potentials (Figure 4(a)). Such effects were observed when the first anti-insect toxin Lqh*α*IT from *Leiurus quinquestriatus hebraeus* scorpion venom was tested on cockroach isolated axon [32, 33]. Later, several new anti-insect toxins with similar Lqh*a*IT characteristics were described, as for example BoT IT1 [23, 24].

*Alpha* toxin (Lqh*α*IT) was also tested on cockroach giant axon in *in situ* conditions. The first observed effect, after the toxin's application, was a repetitive generation of slightly prolonged action potentials in response to short stimulation—such effect has never been observed in the experiments on isolated giant axon (Figure 4(c)). Later on, *plateau* action potential sometimes appeared (Figure 4(d)), but it was never as long in duration as in the case of isolated axon, even after a prolonged application of high toxin concentration. The influence of axonal microenvironment created by glial cells and other axons is a very likely factor in this matter. And it is necessary to consider the fact that the experimental conditions may vary from the physiological ones in a very diverse degree, which in turn may affect significantly the effects of the tested molecules.

 Electrophysiological experiments performed on the cockroach axon helped to define a new group of scorpion neurotoxins: the *alpha*-like toxins. Receptor site of these molecules overlaps with receptor site 3 on sodium channel, where *alpha* toxins bind [18, 19]. *Alpha*-like toxins do not express selectivity toward insect or mammalian sodium channels. Pharmacological characteristics of *alpha*-like toxins are similar but not identical with those of *alpha* neurotoxins. They induce *plateau* potentials, but in addition, they depolarize progressively the axonal membrane. They inhibit the inactivation of sodium current, but to a lesser degree than in the case of Lqh*α*IT. Moreover, a tail current appears, which increases when depolarizing pulses are applied repeatedly (every 5 s) and at the same time, the peak sodium current decreases [18, 19].

Along with the science progressing, modern techniques in molecular biology were applied and a “new era” in scorpion anti-insect toxins launched. One of the most noteworthy approaches towards this issue so far have been presented by Professor Michael Gurevitz and Dr. Dalia Gordon from the Department of Molecular Biology and Ecology of Plants, Tel Aviv University, Israel. They and their collaborators isolated the genetic material responsible for the synthesis of toxins from the scorpion venom, they defined the cDNA sequence and developed artificial expression systems [33–36]. New, recombinant toxins Lqh*α*ITr [36], Lqh IT2 [37] and Bj-xtrIT [38], tested on the cockroach isolated giant axon, showed much resemblance in their mode of action to the native counterparts. Such studies provided an extremely valuable conclusion: the activity of properly prepared recombinant toxins is the same as that of the native ones. This was a huge step in the field of toxinology. Nowadays, the recombinant toxins are accessible in a greater number than the native molecules and the advent of further mutations is feasible. It is pivotal now to study the molecular basis of anti-insect selectivity, as well as anti-mammalian specificity.

Toxicity tests, binding studies and electrophysiological recordings along with the molecular modeling, gene cloning and the site-directed mutagenesis create an opportunity to investigate the molecular basis of toxins' activity. The experiments performed on *alpha*-like toxins from *Buthus martensi* Karsch using cockroach axon showed that mutation of a single amino acid can change completely the toxin mode of action [39]. A phrase: “a story of one amino acid” may well summarize the long history of multiapproach studies on depressant toxins from *Leiurus quinquestriatus hebraeus.* In the experiments on the isolated axon of the cockroach, it has been shown that the replacement of the amino acid in position 58 from Asn to Asp is able to change completely the toxin's mode of action. At the same time, its affinity to sodium channel target and its toxicity decreased. Conclusion from these studies was as follows: Asn in position 58 plays the mandatory role in the activity of depressant toxins [13, 40].

The isolated axon of the cockroach was also used in the tests on the toxins obtained from the spider venom. In most cases they prolonged the duration of action potential, however, *plateau* action potential could only be generated in the presence of a potassium channel blocker. Sodium current inactivation was inhibited in the presence of the toxin, but never to such a degree as in the case of scorpion *alpha* toxins [41–43]. Postapplication of Lqh*α*IT increased the late sodium current recorded during depolarizing pulse (Stankiewicz, personal observations).

## 3. DUM Neurons in Patch-Clamp and Microelectrode Techniques

In 1989 and 1990 two articles describing the application of patch-clamp technique in the study on the activity of neurosecretory dorsal unpaired median (DUM—Figure 1(c)) neurons from terminal ganglion of *Periplaneta americana* nerve cord were published [44, 45]. The technique of single DUM cell isolation and patch-clamp recording on it have been developed in Laboratory of Neurophysiology at Angers University in France by Professor Bruno Lapied. DUM neurons possess an endogenous pacemaker activity which depends on a wide range of ionic membrane conductances [46]. Various types of receptors provide a very precise regulation of the neurons' spontaneous activity and neurosecretory function. DUM neurons represent an outstanding model for studies on intracellular processes [47–49] as well as for pharmacological tests [50, 51]. They have also been used in toxinological experiments.

 There were several experiments on DUM cells performed simultaneously with observations on an isolated axon. Although DUM neurons represent a much more complex model of bioelectrical activity than the axon, the observations were similar and the conclusions-compatible [25, 52]. Background sodium channels (bNa) in DUM neurons were examined using the patch-clamp cell-attached technique [53]. They appeared to be a new target for Lqh*α*IT toxin, even more sensitive than the classical voltage-dependent Na channels in this preparation. The activity of bNa is limited at membrane potential −50 mV (DUM neuron resting potential) and can be “liberated” under Lqh*α*IT action or at very negative (−90 mV) membrane potential. In the presence of the toxin (10^−8^ M), unclustered, brief single channel openings in control (at −50 mV) were transformed into large, multistep amplitude bursting activity, separated by periods of silence. Open probability of the channels increased by about 20-fold. Such channel activity was well corresponding to the transformation observed in DUM neurons: from regular beating, spontaneous activity to rhythmic bursting [53, 54].

 Background sodium channels in DUM neurons are also the target for *beta* toxin (VII) from Brazilian scorpion, *Tityus serrulatus*, venom [55]. In the toxin's presence, single-amplitude openings of the channels were replaced by events with several distinct subconductance current levels. Channel open probability rose by about 50-fold and similarly did the open-time duration; additionally, very long duration events emerged. Classical voltage-dependent sodium channels were also modified in a manner typical for *beta* toxin. The experiments performed with calcium imaging demonstrated the rise in the intracellular calcium concentration in the presence of toxin VII. A very complex study of this phenomenon evidenced the participation of high-voltage activated N-type calcium channels and the activation of noncapacitative calcium entry (NCCE). An important conclusion has been drawn from these studies that the reduction of the activity of NCCE may prove a useful strategy in the development of a drug for antienvenoming therapy [55].

**Figure 5:**
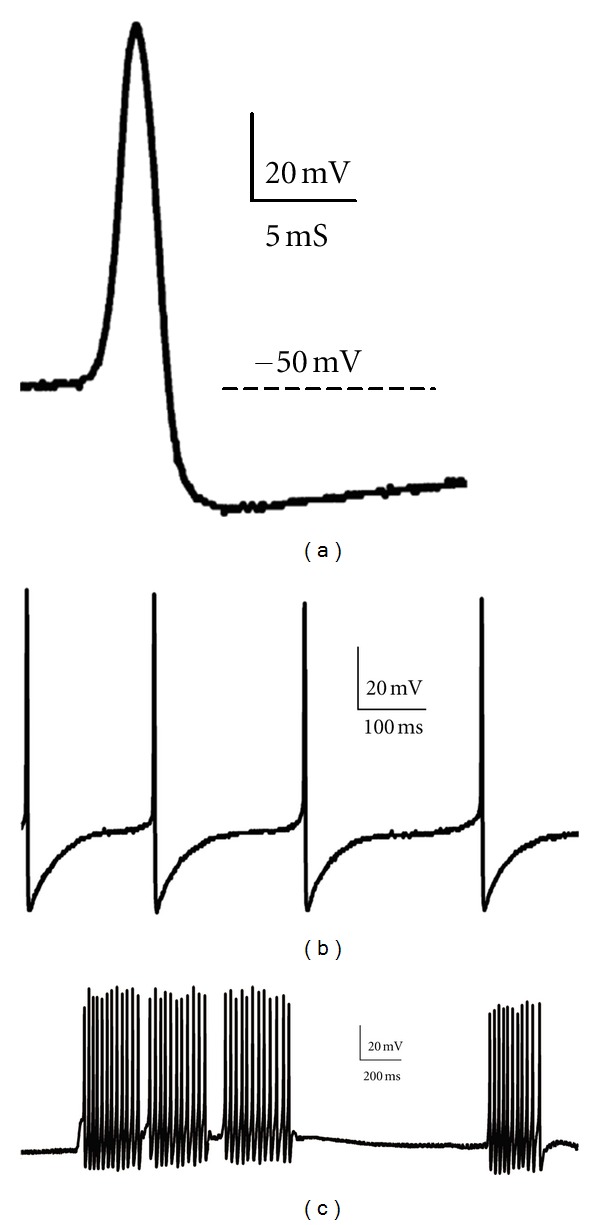
Activity of dorsal unpaired median (DUM) neurons from terminal abdominal ganglion, examined with the microelectrode technique. (a) Single action potential and (b) regular spontaneous activity recorded from the neuron in the ganglion—in *in situ* conditions, in control. (c) Radical change of neuron regular beating discharges into bursting activity in Lqh*α*IT (10^−6^ M) presence.

The activity of DUM neurons can also be observed in *in situ* conditions using the microelectrode technique. Terminal abdominal ganglion is separated from the nerve cord, desheated and fixed. Neurons remain in their place in ganglion surrounded by glial cells and other neurons. DUM neurons are recognized by spontaneous action potentials when the microelectrode enters the cell. The pattern of neuronal activity is similar to the recordings obtained on isolated cells, however, the effect of toxins is not exactly the same. The application of *alpha* toxin (Lqh*α*IT, 10^−5^ and 10^−4^ M) never induced *plateau* action potential *in situ*; only transformation from regular firing into bursting activity was observed and the bursts were generated from a level of slight depolarization (Figure 5(c)). In the ganglion, DUM cells are mainly under the influence of glial cells, as well as other neurons; thus, the ionic microenvironment remains partially undisturbed. In order to investigate the toxin's mechanism of action, isolated neurons should be used by all means, however, *in situ* experiments may in some cases reveal more on physiological effect of toxic molecules. The interactions between two neurotoxins observed on isolated and *in situ* DUM cells are also different (Stankiewicz et al., in preparation).

## 4. Cercal Nerve—Giant Interneuron Synapse and Single-Fiber Oil-Gap Technique

**Figure 6:**
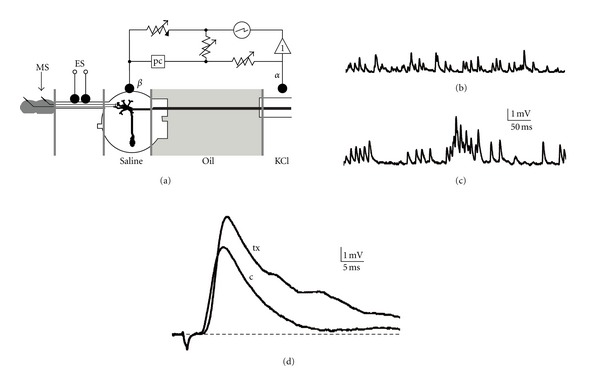
Single-fiber oil-gap method applied for studying synaptic transmission between cercal nerve and giant interneurone. (a) The scheme of electronic circuit used to record postsynaptic events. Recording electrode (*α*) bathed in isotonic KCl solution is connected with high-input impedance, negative capacitance amplifier (1); extracellular electrode *β* is in contact with last abdominal ganglion. Wheatstone bridge is connected to recording circuit and it is used to apply polarizing current (pc) through postsynaptic membrane. The desheated ganglion is superfused with saline or test solution. The dissected axon is bathed in paraffin-oil. MS—mechanical stimulation, ES—electrical stimulation applied to cercal nerve (according to [59, 60]). (b) Control conditions—unitary excitatory postsynaptic potentials (uEPSP) recorded as spontaneous activity of preparation and (d) c—control excitatory compound postsynaptic potential (cEPSP) observed as the effect of cercal nerve electrical stimulation. (c) The increased spontaneous activity (uEPSPs) and (d) tx—cEPSPs observed 10 min after Lqh*α*IT (10^−6^ M) application using pneumatic injection in synapse vicinity.

 The information about mechanical stimulation arising in the cockroach cercal sensory neurons is transmitted to giant interneurons. Cercal nerves X and XI are connected with giant interneurone dendritic tree by inhibitory and excitatory synapses, respectively, located in the last abdominal ganglion. The activity of these synapses can be observed using the single-fiber oil-gap technique (Figure 6(a)), developed by Professor Jean-Jacques Callec in Rennes University [56, 57], later improved and applied for several years by Professor Bernard Hue from Laboratory of Neurophysiology at Angers University, France [58–60]. This method allows the extracellular recording of spontaneous and evoked excitatory postsynaptic potentials (EPSP) as well as inhibitory postsynaptic potentials (IPSP) [57, 60, 61]. Nerve XI is classified as excitatory and its fibers activate nicotinic receptors at postsynaptic membrane. Unitary excitatory postsynaptic potentials (uEPSP) result from the stimulation of single hair mechanoreceptors covering the cercus; composed postsynaptic potentials (cEPSP) are the effect of cercal nerve XI electrical stimulation. At the presynaptic face of such synapses, muscarinic receptors are present, the role of which is to regulate the acetylcholine releasing by negative feedback [62]. Muscarinic receptors were also found on postsynaptic membrane [63]. Stimulation of nerve X evokes inhibitory postsynaptic potentials (IPSP) via activation of GABA receptors [61]. Single-fiber oil-gap method allows to perform long-term experiments (even up to 12 hours) and well-adjusted perfusion of the ganglion enables to obtain reliable dose-response curves. Using the iontophoretic application of acetylcholine or carbachol to postsynaptic membrane vicinity permits to discriminate between pre- and post-synaptic action of the tested drugs. Synaptic preparation from the cockroach represents a remarkably useful model for pharmacological studies, however, obtaining a high-quality stable preparation requires much experience.

Cockroach synaptic model allowed to test the toxins which are active on cholinergic receptors. Snake venom is a well-known source for them. *Alpha*-bungarotoxins and k-bungarotoxins completely blocked uEPSP and cEPSP at concentration of 10^−7^ M. All performed experimental protocols indicated that the toxins block neuronal postsynaptic nicotinic receptors and *α*-bungarotoxin was more effective [64, 65]. Scorpion *alpha* toxin Lqh*α*IT induced a substantial increase in the postsynaptic spontaneous activity, that is, in the frequency and the amplitude of uEPSP (Figures 6(b) and 6(c)), as well as in the amplitude and duration of cEPSP (Figure 6(d)). Higher presynaptic activity results in the increased releasing of acetylcholine, however, it activates the negative feedback via presynaptic muscarinic receptors in turn and after several minutes of the toxin's action, a decrease in postsynaptic events can be observed (Stankiewicz, personal observations).

 The experiments performed using the single-fiber oil-gap method with the scolopendra, *Scolopendra *sp., venom determined its components which induced the depolarization of the cockroach postsynaptic membrane and the decrease in EPSP amplitude. Such effect was limited after pretreatment with atropine. Along with the studies performed on *Drosophila* muscarinic receptors (Dm1) expressed in *Xenopus* oocytes, it was evidenced that the venom of scolopendra comprises a component acting as agonist on insect muscarinic receptors [66].

Lastly, the experiments performed on cockroach synaptic preparation contributed to studies which allowed to explain the mechanism of synergistic interaction between chemical neurotoxins: pyrethroids (permethrin) and carbamates (propoxur) [67]. The conclusions from these studies are important for practical implementation in the field of crop protection.

## 5. Synaptosomal Preparation from Cockroach Nerve Cord

The nerve cords of cockroach (*Periplaneta americana*) have also been used to prepare synaptosomes, which are functional vesicles containing the nervous terminals. The synaptosomal preparation is easy to obtain and has been applied in several pharmacological tests. As illustration, binding assays of many radiolabeled toxins have been successfully characterized with synaptosomes [24, 42, 43, 68]. In addition, studies of photoaffinity labeling using ^125^I TsVII as a ligand in synaptosomes of nerve cord from cockroach indicated for the first time the molecular weight of the scorpion toxin receptor from the insect nervous system which was suggested to be associated with voltage sensitive Na^+^ channels [68]. More recently this preparation was also successful applied in binding studies involving radiolabelled spider toxins acting in sodium channels (De Lima, in preparation). Results obtained from electrophysiological experiments are often completed using synaptosomal preparation coming from cockroach nervous system.

## 6. Summary

Nervous system of the cockroach (*Periplaneta americana*) can be recognized as a remarkably useful model preparation in multiple electrophysiological techniques, which allows to perform pharmacological tests on diverse levels of nervous system organization. Confronting the results obtained reveals that a toxin may affect the activity of the same nervous structure with diverse effects, depending on the experimental conditions and this conclusion should be taken into account prior to any definite statement concerning the mode of action of any toxin.
